# Development and Characterization of Silver Containing Free Standing Polymer FILMS for Dosimetry Applications

**DOI:** 10.3390/polym13223925

**Published:** 2021-11-13

**Authors:** Mantvydas Merkis, Judita Puišo, Diana Adliene, Jurgita Laurikaitiene

**Affiliations:** Department of Physics, Kaunas University of Technology, Studentų str. 50, 51368 Kaunas, Lithuania; matvydas.merkis@ktu.lt (M.M.); jurgita.laurikaitiene@ktu.lt (J.L.)

**Keywords:** silver nanoparticles, polymer composites, X-ray irradiation, radiation dosimetry

## Abstract

Polymer gels and films, due to their near equivalence to biological tissue, are amongst the most promising future dosimetry tools for medical applications. The application of polymer dose gels is limited by the sensitivity of dose readout methods and dose gel properties. It is a challenge to find suitable dosimeters for registration of doses delivered to the target by orthovoltage therapy units. The application of metal-particle-enriched polymer composites for dose registration in X-ray therapy might be an elegant solution, especially if recent dose-reading technologies exploring advantages of different physical phenomena are involved. In this work, X-rays from the orthovoltage therapy range were used for the irradiation of experimental samples. In addition, radiation-induced processes of formation of silver nanoparticles in AgNO_3_–PVA gels and in free standing AgNO_3_PVA films, also containing some additional solvents, namely glycerol, ethanol, and isopropanol, have been investigated, with the aim to apply the developed composites for medical dosimetry purposes. A simple and environmentally friendly method for the formation of free-standing AgPVA films at room temperature was proposed and realized for preparing AgPVA films for investigation. Radiation-induced synthesis of silver nanoparticles in AgPVA composites was investigated, analyzing LPSR-based UV-VIS spectral changes to the irradiated films with respect to irradiation doses, and dose-related tendencies were also evaluated. It was shown that AgPVA films were more sensitive for detection of doses from the interval 0–1.0 Gy, thus indicating potential application of AgPVA films for dosimetry purposes.

## 1. Introduction

Assessment and control of irradiation doses to patients during radiotherapy treatment or radiological examination is a top priority in the medical radiation field. However, registration and monitoring of the occupational exposure to the staff dealing with ionizing radiation is another important issue. Due to this dosimetry and related materials, instruments and methods have become a prominent research field in physics and chemistry as it involves different radiation-induced processes and phenomena in irradiated materials: thermoluminescence (TLD) [1,2,3], optically stimulated luminescence (OSL) [3,4,5,6], electron spin resonance (ESR) [7,8], radioluminescence (RL) [9], and others. Dosimetry methods utilizing the above-mentioned phenomena are well established, when speaking about high-energy and high-dose irradiation; however, dose registration in the interval 0–0.1 Gy might be challenging. With the development of new radiation treatment and diagnostic modalities, the interest in new dosimetry methods and techniques, and especially advanced materials with improved capability for radiation registration, has grown rapidly during the last decade [1,3,4,10]. 

The application of silver (Ag) and gold (Au) metallic nanoparticles as additives to detector materials is a possible solution to increase detector’s sensitivity and relative high-photon absorption. Having large active surface and relatively high photon-absorption ability (of metals), these particles may absorb a large amount of energy (which is related to absorbed dose) at the lower irradiation level as compared to the bulk material of a radiation detector. Moreover, local surface plasmon resonance (LSPR) properties of metal nanoparticles may also aid radiation detection, since the intensity of plasmonic peaks increasing linearly with the absorbed dose [11,12]. The specific interest in metal nanoparticles is also caused by their optical properties, which strongly depend on the particle size, shape, and surrounding media. 

Metal nanoparticles could potentially be applicable in dosimetry techniques such as OSL, TL, and RL. Usually, bulk gold indicates high absorption throughout the completely visible range, except for a slight dip around 400–500 nm [13,14]. However, due to the reduction of Au particle size from 500 nm to <10 nm, the gold colour changes from orange to red due to the occurrence of the surface plasmon resonance [13,14]. Free electrons of Au, Ag, Co, and some alkali metals are responsible for the induction of the LSPR in the visible light region [14]. Changes to the structure of the medium containing dispersed nanoparticles lead to changes in the relevant optical properties of the dispersed medium [13,14]. The refractive index of a dispersed medium and the average distance between neighbouring metal nanoparticles influence the shape of the UV-VIS spectrum. Two main options are explored when fabricating metal particles containing materials intended for radiation dosimetry purposes: nanoparticles can be added and dispersed in the bulk material, or nanoparticles can be synthesized from metal salts by applying different techniques and left in the host matrix. 

One of the common techniques for obtaining metal nanoparticles is radiolysis. This technique is easily controlled and adaptable, environmentally friendly, and fast; it avoids the use of harsh reducing agents and it does not introduce impurities into the medium [15,16,17,18]. A radiolysis technique is used to generate nanoscale metals and nanocomposites at room temperature and normal pressure. The ionizing radiation transfers energy into the medium through various processes, among which the most prominent processes are ionization and excitation of atoms, the breaking of chemical bonds, cross-linking, the disintegration of molecules, etc. Exposure of metal salt solution to ionizing radiation produces active radiolysis products (hydrated electron ($e_{\mathrm{aq}}^{-}$), hydrogen atom ($H^{}$), and hydroxyl radical ($OH^{-}$), and other organic radicals may also be produced) that reduce metal ions from the salt and form atomic nanoparticle seeds [15,16,17]. 

Radiation-chemical gain, i.e., number of active, emerged, disintegrated, and recombined particles (molecules, ions, radicals, etc.) per 100 eV of absorbed energy, depends on a diversity of physical and chemical factors and is a function of absorbed radiation dose and varies within a wide range (from $10^{-6}$ to $10^{8}$) [18]. However, silver ions reduced from salt by ionizing radiation may easily agglomerate, if there is no any protection [19,20,21]. 

Stabilizers such as agar, gelatine, polyvinylpyrrolidone (PVP), polyvinyl alcohol (PVA), and surfactants are used to prevent nanoparticle agglomeration in the colloids. However, if a sufficient amount of energy is transferred to polymer molecules, ionization and excitation may occur. Depending on irradiation conditions, this may result in polymer chain crosslinking or chain scission reactions, thus influencing the initial properties of polymer medium thought to host of the reduced nanoparticles. Synthesized metal particles may obtain different shapes, which depend on the colloid preparation method, irradiation parameters, and used stabilizers [22,23]. The shape of the synthesized nanoparticles is also related to the synthesis reaction rate. Spherical metal particles with the minimum surface for a given volume are obtained by the reduction of one-capacity silver ions under controlled thermodynamic conditions [22]. The reaction rate of semi-spherical nanoparticles is ~4 times smaller than cubic nanoparticles but ~3.5 times higher than triangular nanoparticles [24].

Typically, the rate of formation for reducing species by gamma radiation can be tuned continuously from the $\mu \mathrm{mol}\cdot L^{-1}h^{-1}$ to the $\mathrm{mol}\cdot L^{-1}h^{-1}$ range [25]. The dose rate is a main parameter governing the complex nucleation−growth reactions of nanoparticles [26]. During irradiation of silver salt solution with presence of PVA by a low gamma irradiation’s dose rate, the initial growth rate of silver is slow, because the capping rate dominates and small spherical nanoparticles are formed. By increasing the gamma irradiation dose rate, the rapid adsorption of cross-linked PVA chain on newly formed silver nanoparticles leads to formation of triangular nanoplates [27,28]. The X-ray irradiation is a possible competitor to traditional gamma processing. The primary interaction mechanism with matter is identical. The secondary electrons from Compton scattering initiates ionization events and activates a chemical reaction. B. Croonenborghs and co-workers [29] investigated the differences in modification of polymers (polyethylene (PE), polypropylene (PP), polystyrene (PS), plasticized polyvinylchloride (PVC), and an acrylonitrile–butadiene–styrene copolymer (ABS)) caused by gamma- and X-ray irradiation and concluded that radiation-induced effects in polymers were very similar for both types of radiation. Provided conclusions were based on the fact that gamma and X-rays being low-LET (Linear Energy Transfer) ionizing radiations similarly govern the radiolytic yields of species reduced at the same radiation penetration depth [30].

Dose-dependent changes in easily assessable properties (changes in UV-VIS, Raman, FTIR spectrum) presuppose provision that polymer–metal salt solutions and especially dried and solidified forms of these solutions (films) could be used for radiation detection purposes. However, there is a lack of information regarding fabrication, investigation, and characterization of such free-standing polymer-metal films. 

The aim of this work was the development and characterization of nanoparticle-enriched radiation sensitive free standing Ag/PVA films, which can be utilized for dosimetry purposes in orthovoltage radiotherapy.

## 2. Materials, Instruments, and Methods

Chemical composition of the proposed polymer composites, manufacturing technology of nanoparticle enriched free-standing films, sample irradiation conditions, and materials characterization methods are discussed in this section.

### 2.1. Materials

Paying attention to the requirements of the green economy, radiation sensitive silver (Ag)-enriched polyvinyl alcohol (PVA) hydrogels have been developed for dose registration in medical field. PVA was chosen as a hosting material for Ag nanoparticles synthesized via radiolysis of silver nitrate due to the fact that it is water-soluble polymer characterized by excellent thermo-stability, chemical resistance, and hydrophilic properties; it provides excellent thin film forming properties and is optically transparent. In addition, this biodegradable and biocompatible synthetic polymer is well known for its low cytotoxicity. In order to prevent oxidation of synthesized Ag particles, some solvents, e.g., glycerol, ethanol, and isopropanol, were used as additives to the hydrogels.

Silver nitrate (${\mathrm{AgNO}}_{3}$, purity ≥ 99.0%, Sigma-Aldrich Chemie GmbH, Regensburg, Germany), (Poly(vinyl) alcohol, ${\left(C_{2}H_{4}O\right)}_{n}$, M_w_ ~ 31,000, Sigma-Aldrich Chemie GmbH, Regensburg, Germany), glycerol ($C_{3}H_{8}O_{3}$ purity > 99.5%, Euro-Chemicals GmbH, Nordhorn, Germany), ethanol ($C_{2}H_{5}\mathrm{OH}$, purity ~ 96%, Euro-Chemicals GmbH, Nordhorn, Germany), and isopropanol (${\mathrm{CH}}_{3}{)}_{2}\mathrm{CHOH}$), with purity ≥ 99.0%, Euro-Chemicals GmbH, Nordhorn, Germany) were used for hydrogel formation.

Two types of samples were prepared: hydrogels in liquid form and free standing gel films with reduced amount of water after drying.

#### 2.1.1. Formation of Ag-Enriched PVA Hydrogels 

In order to investigate nanocomposites’ (gels’) ability to detect low-energy and low-dose radiation, silver-enriched PVA-based hydrogels have been produced as follows: PVA powder was dissolved in the distilled water under continuous stirring. A constant temperature of 60 °C was kept during the entire stirring process. Later, PVA solution was cooled down to room temperature. A certain amount of ${\mathrm{AgNO}}_{3}$ was added drop-by-drop to the solution. Two sets of AgPVA gels with different silver nitrate concentrations were prepared. The increased gel viscosity and prevention of oxidation of the synthesized silver particles were realized by adding some amount of glycerol instead of water to the initial AgPVA solutions forming new AgGlyPVA gels while keeping the same concentrations of AgNO_3_ as there were in the initial gels. In order to avoid metallic silver reduction by daylight, freshly prepared polymer gels were poured into standard polystyrene (PS) cuvettes and kept at room temperature (20 °C) in the dark for 72 h. Reference samples of pure PVA water solution and PVA + Glycerol were prepared as well. Initial PVA solutions were colour-less and transparent. Chemical composition of the prepared solutions is provided in Table 1.

#### 2.1.2. Formation of Ag Enriched Free Standing PVA Films

Gel solutions for the formation of free-standing Ag-enriched PVA films were prepared using the same procedure as was described in the previous section. In order to assess the effectiveness of additional solvents in preventing synthesized Ag from undergoing oxidation, amounts of glycerol, ethanol, or isopropanol were added drop by drop to the gel solutions. Prepared gel solutions were casted in Petri dishes forming thin layers and were left to dry at room temperature (20 °C) in dark for 72 h. In order to obtain flexible films, a series of samples were prepared by varying AgNO_3_ concentrations and adding different solvents (glycerol, ethanol, and isopropanol) to initial gel’s solution. The initial PVA-AgNO_3_ water solutions with or without additional solvents were colourless and transparent; however, during the drying and water evaporation process, the colour of samples changed to light yellow. The colour intensity was dependent on AgNO_3_ concentration in the gel. Slight discolouration of as-prepared gels was attributed to the possible formation of silver seeds during the drying period and their aggregation to silver nanoparticles, which were stimulated via interactions between silver ions and PVA due to the decreasing amount of water in samples. Reduction in water amount during the entire drying process was also responsible for the reduced adhesion force between the gel layer and the surface of the Petri dish. This led to the blistering effects in the gel through which modified Ag particle-enriched PVA free-standing film were formed. Dried samples were slightly heated and removed from Petri dishes, thus making free-standing gel films ready for investigation. Chemical compositions of the prepared AgPVA gel films are provided in Table 2. 

Measured thickness of the obtained films varied between 0.098 and 0.113 mm.

#### 2.1.3. Irradiation and Analytical Techniques

Both types of AgPVA gels (liquid and films) were irradiated in X-ray therapy unit GULMAY D3225 (GULMAY GmbH, Krefeld, Germany). X-ray energy (140 keV) and doses (up to 8 Gy) were chosen for irradiation of samples in accordance with the main aim of this research: to investigate the radiation sensitivity of the Ag-enriched PVA gels and to assess polymer gels’ capability to detect low energy and low dose irradiation, which is relevant for orthovoltage radiotherapy. 

Optical properties of experimental samples were investigated using UV-VIS spectrometer Ocean Optics with USB400 (Ocean Optics, Inc., Dunedin, FL, USA); wave range 190–900 nm, resolution—1.5 nm).

Radiation-induced changes in the molecular structure of polymer gels were analyzed using Raman microscope inVia (JF RENSHAW LTD, Liverpool, UK), equipped with 532 nm wavelength diode laser and providing 0.3 mW power at the sample surface. 

Estimation of polymer gel’s radiation sensitivity and sample’s dosimetric evaluation was based on the analysis of radiation induced changes of sample’s optical properties related to radiolysis of Ag particles and polymerization mechanisms in PVA gels containing silver nanoparticles.

### 2.2. Radiolysis and Polymerization Mechanisms in the Irradiated Samples

The process of particle growth usually occurs through the Oswald ripening mechanism. As a result, the particle size increases continuously because the larger particles grow on account of the dissolution of smaller ones [31]. A convenient procedure to restrict their growth is the in situ synthesis of nanoparticles within the polymer matrix with an improved architecture, i.e., within the three-dimensional network of hydrophilic polymers. 

Assuming that a 3D network of hydrogels is a suitable template for the in situ synthesis, stabilization, and distribution of metal nanoparticles [32,33,34,35,36], the synthesis of Ag nanoparticles incorporated into a PVA hydrogel matrix was initiated by an ionizing-radiation-induced radiolysis process, and inorganic/organic hybrid cross-linked nanocomposites were formed.

If the concentration of AgNO_3_–PVA in aqueous solution is relative small, the radiation energy is mainly absorbed by the solvent, causing predominant water radiolysis and generation of primary radicals, most important of which are ($e_{\mathrm{aq}}^{-})$, hydroxyl radicals, and hydrogen atoms. Hydrate electrons ($e_{\mathrm{aq}}^{-})$ and hydrogen ($H^{\cdot }$) stimulate reduction of silver ions (${\mathrm{Ag}}^{+})$ to neutral silver atoms (${\mathrm{Ag}}^{0}$). A large number of neutral silver atoms (${\mathrm{Ag}}^{0}$) could be produced in the gel because the electron capture cross-section for ${\mathrm{Ag}}^{+}$ is high. The following reactions take place [31]:

(1) $${\mathrm{Ag}}^{+}+e_{\mathrm{aq}}^{-}\to {\mathrm{Ag}}^{0}.$$

(2) $${\mathrm{Ag}}^{+}+H^{\cdot }\to {\mathrm{Ag}}^{0}+H^{+}.$$

The ${\mathrm{Ag}}^{0}$ atoms can join with the excess ${\mathrm{Ag}}^{+}$ ions and produce ${\mathrm{Ag}}_{2}^{+}$ ions. This phenomenon leads to the formation of silver nanoparticles in the water-based gel by the following reaction:

(3) $${\mathrm{Ag}}^{0}+{\mathrm{Ag}}^{+}\to {\mathrm{Ag}}_{2}^{+},$$

(4) $${\mathrm{Ag}}_{n-1}+{\mathrm{Ag}}^{+}\to {\mathrm{Ag}}_{n}^{+},$$

(5) $${\mathrm{Ag}}_{n}+{\mathrm{Ag}}^{+}\to {\mathrm{Ag}}_{n+1}^{+}$$

(6) $${\mathrm{Ag}}_{n+1}^{+}+e_{\mathrm{aq}}^{-}\to {\mathrm{Ag}}_{n+1}$$

(7) $${\mathrm{Ag}}_{n+1}^{+}+H^{\cdot }\to {\mathrm{Ag}}_{n+1}+H^{+}$$

Hydroxyl radical (${\mathrm{OH}}^{\cdot }$) prevents the silver ion (${\mathrm{Ag}}^{+}$) from reduction or induces ion (${\mathrm{Ag}}^{+}$) oxidation. Hydroxyl radical (${\mathrm{OH}}^{.}$) can oxidize the neutral metal atoms by the following reactions:

(8) $$\mathrm{Ag}+{\mathrm{OH}}^{\cdot }\to {\mathrm{Ag}}^{+}+{\mathrm{OH}}^{-}$$

(9) $${\mathrm{Ag}}_{n}+{\mathrm{OH}}^{\cdot }\to {\mathrm{Ag}}_{n}^{+}+{\mathrm{OH}}^{-}$$

In the presence of alcohol in the gel, the (${\mathrm{OH}}^{.}$) and ($H^{\cdot }$) radicals abstract hydrogen from the alcohol [37,38,39] to produce an alcohol radical by following reactions [26,40]:

(10) $$2\mathrm{PVA}\left(H\right)+2{\mathrm{OH}}^{\cdot }\to 2{\mathrm{PVA}}^{.}+2H_{2}O$$

(11) $$2\mathrm{PVA}\;+2H^{\cdot }\to 2{\mathrm{PVA}}^{.}+2H_{2}$$

(12) $$2{\mathrm{PVA}}^{.}\to \mathrm{PVA}-\mathrm{PVA}\;\left(crosslinked\;polymer\right)$$

It is well known that the radiation crosslinking of PVA molecules is mainly induced by ${\mathrm{OH}}^{\cdot }$ [41,42,43]. However, different crosslinking routes are possible, as was shown by Ulanski et al. (1994) [41]: the hydrogen bond formation between chains increases the probability of the polymerization mechanism to undergo intra-molecular “recombination” crosslinking rather than inter-molecular “disproportionation.” In the case of oxygen gas, “disproportionation” reactions will predominantly lead to the formation of carbonyl groups. In the presence of oxygen, the PVA radicals will be converted into corresponding (HOO•) peroxyl radicals leading to strand breakage [43].

It should be mentioned that radiolysis of alcohol also produces $H^{+}$ and $e^{-}$ by the following reaction: [38]

(13) $$\mathrm{RHOH}\to \mathrm{RO},{\;e}^{-},{\;H}^{+}$$

which indicates that the reduction of ${\mathrm{Ag}}^{+}$ ions can also proceed through the polymeric radicals ${\mathrm{PVA}}^{.}$:

(14) $${\mathrm{Ag}}_{2}^{+}+e_{\mathrm{aq}}^{-}/{\mathrm{PVA}}^{.}\to {\left(\mathrm{Ag}\right)}_{n}.\;$$

Solvated electrons produced during the irradiation of AgPVA liquid gel react rapidly and contribute to the production of crosslinks in PVA [35]. The radicals are produced in a very short time (10^−12^–10^−6^ s), which indicates that the free radicals could react within ≥10^−6^ s in liquids. The free radicals are relatively stable in a solid PVA polymer and pose crosslinking via slow (seconds) radical migration in order to produce dimerization. The crosslinking of PVA exhibits absorption maxima at around 500 nm wavelength in the UV-VIS spectrum at room temperature. Ag atoms and clusters (${\mathrm{Ag}}_{2}^{+},{\;\mathrm{Ag}}_{3}^{2+},{\;\mathrm{Ag}}_{4}^{2+}\;$) synthesized in a PVA solution exhibit much stronger reducing properties than bulk metal and may react with the surrounding medium undergoing back-oxidation to ions. Due to the ability of various –OH groups in the PVA polymer network to absorb metal ions through secondary bonds and steric entrapment, the formed PVA polymer network prevents the formation of metal hydroxide clusters by hydrolysis of metal ions and also formed nanoparticles from aggregation.

## 3. Results and Discussion

### 3.1. Characterization of the Irradiated PVA Hydrogels

**Figure 1:**
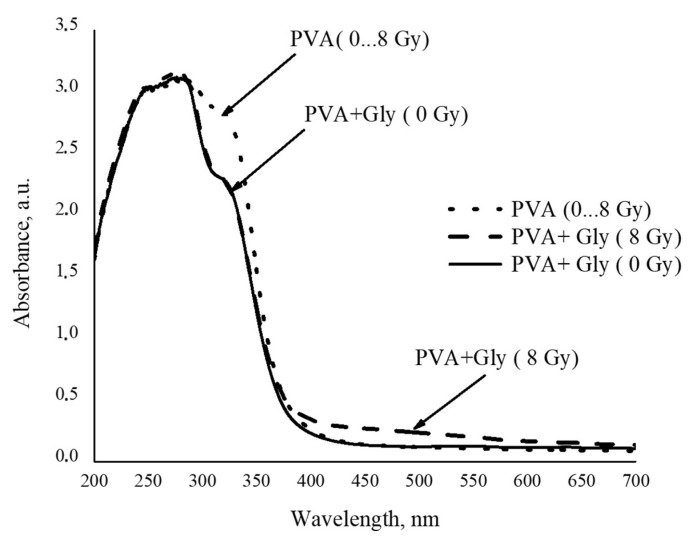
UV-VIS absorbance spectra of PVA and PVA + Gly samples before and after their irradiation with X-ray photons up to 8 Gy.

Initial water solutions of PVA and PVA + Glycerol were colourless and transparent. Irradiation of samples with X-ray doses up to 8 Gy did not show any significant changes in colour or transparency, as can be seen from the UV-VIS absorbance spectra provided in Figure 1.

Two absorption peaks at 279 nm and 322 nm wavelength corresponding to pure PVA [44] were found in UV-VIS spectra of all investigated PVA and PVA + Gly samples. However, no other changes related to irradiation of PVA samples up to 8 Gy were observed regardless of irradiation dose. Addition of glycerol to the PVA solution led to a decrease in the intensity peak at 324 nm wavelength in all investigated PVA + Gly samples, and a new very broad but still detectable radiation-induced absorption band occurred at the wavelength of 493 nm. Observed spectral absorption intensity changes were explained by the X-ray-irradiation-induced crosslinking of glycerol radicals with PVA. Radiation-induced crosslinking was not observed in pure PVA samples.

Two sets of both PVA and PVA + Gly hydrogel samples, containing different concentrations of AgNO_3_, were prepared using procedure described in the Materials, Instruments, and Methods section. The investigation aimed for the assessment of the role of silver salt concentration on the effectiveness of radiation-induced reduction of silver particles. It is known [45,46] that the presence of Ag particles may increase the X-absorption ability of polymer composite, making it an attractive material for medical dosimetry applications. 

**Figure 2:**
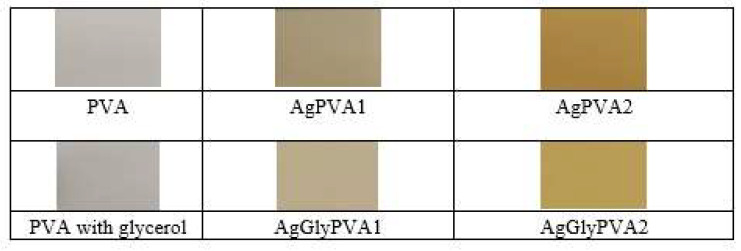
PVA, AgPVA, and AgGlyPVA films after irradiation with 5 Gy dose.

X-ray irradiation of samples led to their colour changes from almost colourless to pale yellow or golden brown due to the reduction of silver ions to neutral silver atoms upon irradiation (Figure 2). 

**Figure 3:**
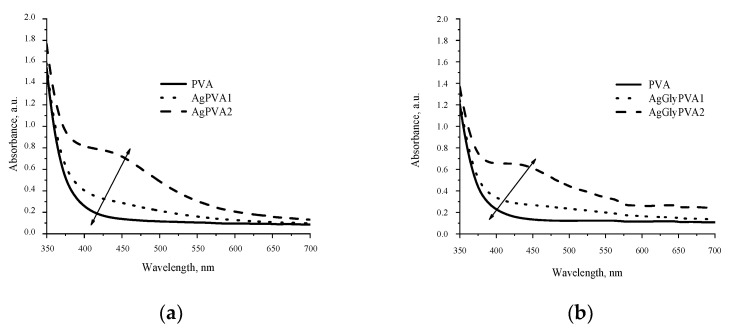
UV- VIS spectra of X-ray-irradiated gels containing two different concentrations of AgNO_3_: (**a**) without additives (AgPVA) and (**b**)with glycerol (AgGlyPVA). Both types of gels were irradiated with 5 Gy dose.

By analyzing UV-VIS spectra of the X-ray-irradiated samples, it was found that all silver-salt-containing alcohol solutions indicated increased absorption intensity in the whole visible light range (Figure 3). The appearance of the characteristic absorbance band in the visible region (450–500 nm) was attributed to the local surface plasmon resonance (LSPR) phenomena caused by the presence of silver nanoparticles in investigated samples, thus indirectly confirming the synthesis of silver nanoparticles in X-ray-irradiated samples. 

The intensity of the LSPR band in irradiated samples was growing with the increasing concentration of AgNO_3_ and tended to shift slowly towards the higher wavelength with the higher dose of irradiation.

**Figure 4:**
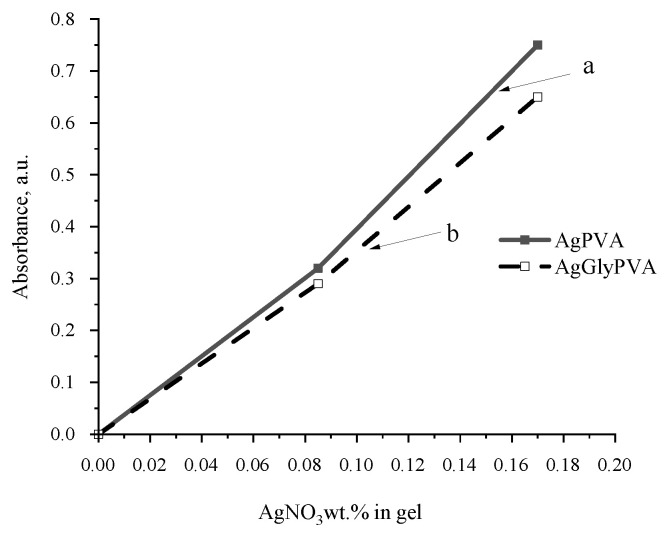
The influence AgNO_3_ concentration on LSPR intensity in irradiated PVA composites: (a) without glycerol (AgPVA); (b) containing some amount of glycerol (AgGlyPVA).

A more detailed comparison of the light absorption ability of gels containing some amount of glycerol and those without revealed that the light absorption was more intensive in samples without glycerol additives (Figure 4), but the absorbance band in samples containing glycerol was shifted towards a higher wavelength. In general, the LSPR intensity, which is dependent on the number of synthesized Ag particles, was almost proportional to the initial concentration of AgNO_3_ in as-prepared PVA samples.

The red shift of LSPR band with the increased amount of silver salt in the initial PVA samples indicates that with the increased silver ion concentration, silver nanoparticles that are dispersive in size are produced and particle agglomeration is possible upon irradiation. Addition of glycerol contributes to the damping and stabilization of the particle-formation process; however, synthesized particles are smaller. 

### 3.2. Characterisation of the AgPVA Free Standing Films

Several sets of free standing composite films with and without additives were formed by casting PVA water solutions in Petri dishes, as was described in the Materials, Instruments, and Methods section. Prepared samples were left for 72 h to dry at room temperature.

It is known [47] that the formation of PVA composite films containing Ag nanoparticles (AgPVAF) is related to the interactions of ${\mathrm{Ag}}^{+}$ ions with radical intermediates of the PVA (especially OH groups) and formation of ${\mathrm{Ag}}^{0}$ [48]. This means that silver nanoparticles are created inside PVA when hydrated electrons are released due to reduction of ${\mathrm{Ag}}^{+}$ ions to ${\mathrm{Ag}}^{0}$. Spontaneous protonation of PVA, which may occur in the AgPVA solutions during the drying process or radiation-induced protonation in irradiated AgPVA films, may lead to the production of cationic intermediates. These cationic intermediates can react with the neighbouring PVA molecules and initiate the crosslinking of PVA acting as a host matrix for the formatted silver nanoparticles. Irradiated and cross-linked PVA is insoluble in water [26].

In order to investigate X-ray radiation-induced processes in free standing PVA films, pure PVA samples, and PVA samples containing different concentrations of AgNO_3_ (0.21wt.% and 1.01 wt.% in the initial solution) were irradiated with doses up to 5.0 Gy, starting with very low doses (0.1 Gy), which are more realistic for medical staff working in nuclear medicine departments. Visual inspection of irradiated films showed that PVA films remained colourless and transparent after irradiation; however, the colour of irradiated AgPVA films changed from light yellow, which was typical for as-prepared silver containing dried samples, to yellow-brown. Glycerol and other additives were responsible for film darkening from yellow to dark brown. It should be noted that at irradiation doses ≥ 2 Gy, the precipitation and fallout of neutral silver atoms were observed on one of the film surfaces (Tollens’ test, also known as silver-mirror test).

**Figure 5:**
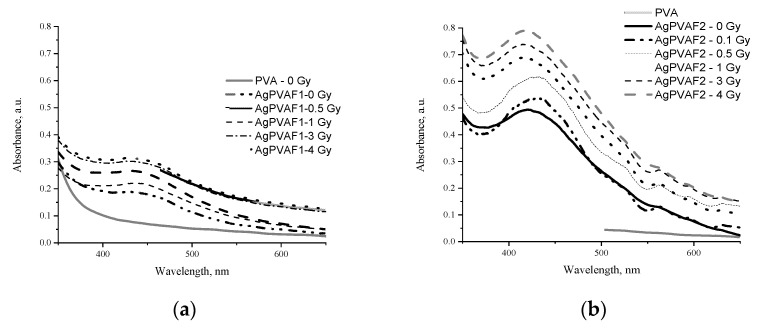
UV-VIS absorption spectra of experimental films containing different concentrations of AgNO_3_ before and after irradiation: (**a**) AgPVAF1 (0.21 wt.%) and (**b**) AgPVAF2 (1.01 wt.%). Spectrum of pure PVA film is provided for the comparison.

UV-VIS absorption spectra of irradiated AgPVAF films containing different amounts of silver ion precursors are provided in Figure 5.

The presence of LSPR peaks indicating formation of silver nanoparticles in PVA was clearly seen in the UV-VIS spectra provided. LSPR peak positions were characteristic for the formed spherical Ag particles. In our case, silver precursors required for the formation of Ag nanoparticles in AgPVA composites were already created in the initial step of film preparation, namely drying of gels at room temperature. LSPR peaks for not-irradiated AgPVAF1 and AgPVAF2 films were observed within the wavelength range of 427–436 nm; their intensity was dependent on the amount of AgNO_3_ in primary samples. In general, the intensity of LSPR peaks was dependent on the concentration of silver precursor and on X-ray irradiation dose in our investigation. 

The LSPR peak at 436 nm was found for AgPVAF1 films containing small amounts of AgNO_3_ (0.21 wt.%) and varied between 416 nm and 427 nm in wavelength for AgPVAF2 films containing 1.01 wt%. The LSPR peak observed for AgPVAF2 films was more intensive. Observed blue shift of LSPR peak and intensity changes with the increasing irradiation dose indicated that X-ray radiation induced the transformation of Ag particle grains, which were produced during the film-drying process for silver nanoparticles and also growth of new silver nanoparticles on the seeds’ surface by silver ions from the former PVA film. The formation of silver nanoparticles in the films was also approved by visual inspection of colour changes in the irradiated films.

**Figure 6:**
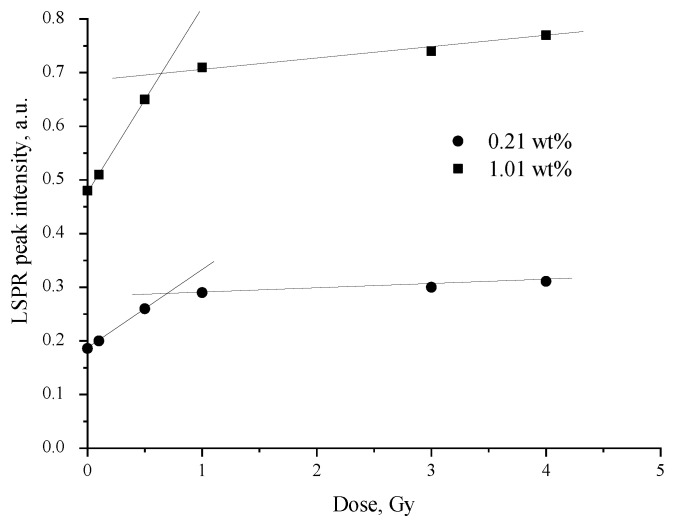
LSPR peak intensity variations with the irradiation dose in AgPVAF films containing different concentrations of AgNO_3_.

Indicative differences between LSPR peak intensity in the range up to 1.0 Gy (estimated dose slope—0.34 Gy^−1^) was observed (Figure 6) for AgPVAF2 films as compared to the interval of higher irradiation doses, indicating a less intensive dose slope of 0.15 Gy^−1^. The LSPR peak values were derived from Figure 6. The similar tendencies were also observed for the AgPVA1 films containing lower concentrations (0.21 wt%) of AgNO_3_: the dose slope in the dose range up to 1.0 Gy was 0.2 Gy-1, and that for the higher irradiation doses was 0.01 Gy^−1^.

For this reason, further investigation of Ag particle formation in X-ray-irradiated AgPVA films was performed in the dose interval of 4.0 Gy, focusing on the radiation-induced changes in low-dose-irradiated films. 

The results of the performed investigations showed the reasonable dose sensitivity of analyzed films in the range up to 1.0 Gy, indicating the potential of these films for medical dosimetry applications. 

**Figure 7:**
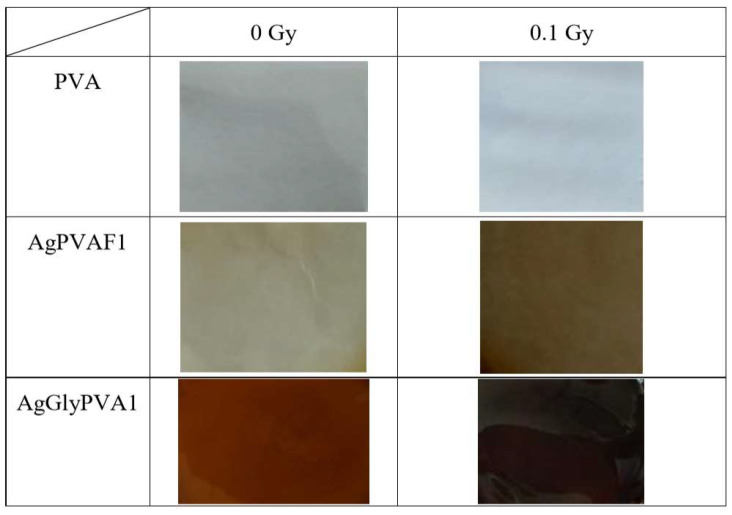
Morphology of PVA, AgPVAF1, and AgGlyPVAF1 films.

Considering film flexibility as the advantageous feature for dosimeters, some amount of water in hydrogels was replaced by glycerol, ethanol, or propanol. It is known that the selection of solvents should be based on the compatibility of their physical and chemical characteristics with the properties of the initial polymer. However, despite the fact that the most important physical characteristics (refractive index and physical density) of glycerol were found to be close to those of PVA, ethanol and propanol were also used as AgPVA film additives when investigating radiation-induced changes in AgPVA films (Figure 7). 

**Figure 8:**
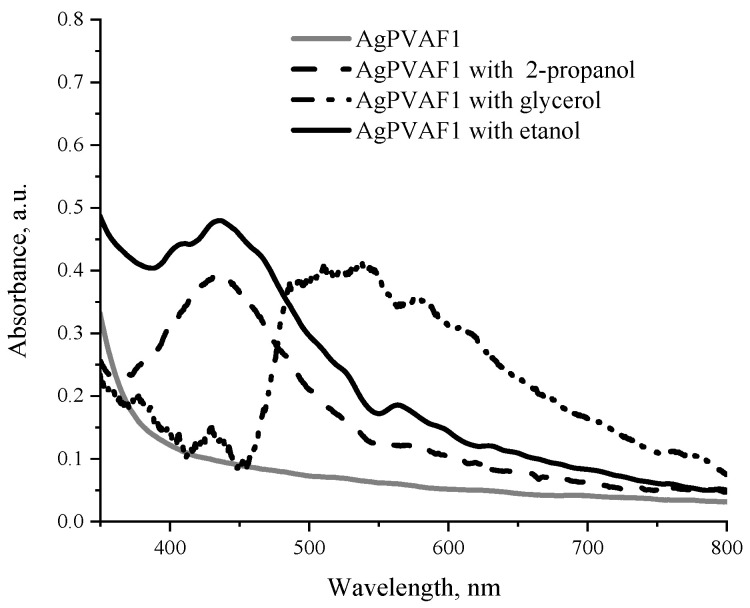
UV-VIS spectra of AgPVAF1 films containing 0.21 wt.% of AgNO_3_ and glycerol (AgGlyPVAF1), ethanol (AgEtaPVAF1), or isopropanol (AgIzoPVAF1) additives and irradiated with 4 Gy doses.

UV-VIS absorption spectra of the AgPVAF1 films containing different additives and irradiated with a 0.1 Gy dose are shown in Figure 8.

The LSPR peak of Ag nanoparticles was observed at 436 nm for both ethanol and propanol additives containing AgPVA films, indicating a slightly higher absorption ability of films containing ethanol. However, exposed glycerol-containing films have shown relatively low intensity of LSPR peak at 429 nm and remarkable increase in the intensity in the wavelength region of 450–600 nm, with a maximum around 500 nm. This may indicate intense-radiation-induced interaction of glycerol with PVA and PVA network formation, leading to expelling of Ag particles from the network. The later suggestion is partially approved by the fact that one of two surfaces of irradiated films became covered by neutral silver atoms (silver mirror test) after irradiation. Unlike the glycerol, presence of ethanol and isopropanol in PVA films stabilizes Ag nanoparticles formation in PVA matrix.

**Figure 9:**
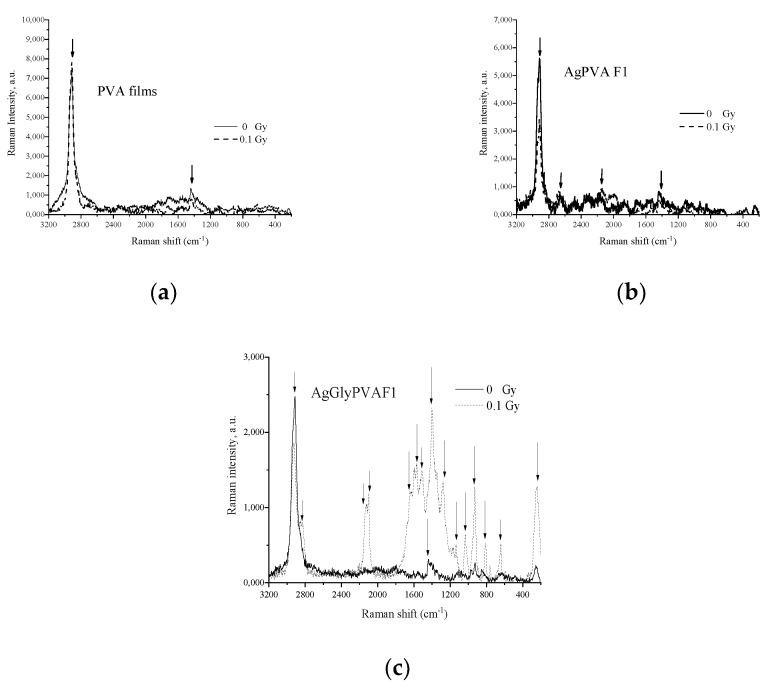
Raman spectra of PVA based films: (**a**) PVA, (**b**) AgPVAF1, and (**c**) AgGlyPVAF1 before and after X-ray irradiation (dose 0.1 Gy).

More details on molecular structure formation in X-ray-irradiated PVA-based films was obtained by investigating Raman spectra of irradiated and not-irradiated experimental samples PVA, AgPVAF1, and AgGlyPVAF1provided in Figure 9.

It can be seen that spectra of PVA films before X-ray irradiation showed only two strong scattering peaks of PVA at 1434 cm^−1^ and at 2915 cm^−1^, corresponding to the bending mode of C–H and O–H mode and symmetric bending mode of the C–H group [39,49,50,51,52]. X-ray exposure was responsible for the decreased intensity, narrowing, and shift of both Raman peaks, indicating some small rearrangements in PVA structure related to the initiation of networking issues. Weak Raman peaks were found at 2172 cm^−1^ and 2653 cm^−1^ [39,49,50,51,52], related to the formation of Ag particles found in Raman spectra of AgPVAF1 samples, as well as the most important peak at 1532 cm^−1^, which corresponds to PVA’s hydroxyl group and typically indicates interaction between PVA molecules and the silver nanoparticles [39,40,41,42,43,53]. After the irradiation peak, a more intense sharp corresponding peak was detected at 1540 cm^−1^, indicating changes in the film due to formation of Ag particles and their accommodation in the PVA matrix. It should be noted that in general, X-ray exposure of AgPVAF1 films contributed with the overall decrease in the Raman intensity. 

Raman spectra of AgGlyPVAF1 showed the main peaks at 2916 cm^−1^ and 1440 cm^−1^, corresponding to the stretching mode of C–H and symmetric bending mode of C–H group of PVA and glycerol. Additional peaks appeared after sample irradiation in the low-frequency region of the Raman spectra at 256 cm^−1^, 639 cm^−1^, and 926 cm^−1^ and were attributed to wagging and stretching modes of the O–H group in PVA and glycerol, respectively [49,50,51,52]. Due to the embedding of Ag nanoparticles in PVA, the Ag–N vibrational band at 245 cm^−1^ was reduced. The stretching vibration band of the nitrate ion appeared at 1045 cm^−1^ [39,41,42,43,44,53]. Comparison of Raman spectra of AgPVAF1 films (Figure 9b) and Raman spectra of AgGlyPVAF1 films containing glycerol additives (Figure 9c) after X-ray exposure to 0.1 Gy shows that a number of easily detectable additional peaks appeared in irradiated AgGlyPVAF1 samples: unassigned Raman peaks at 2119, 2099, and 1598 cm^−1^ and peaks at 1573 cm^−1^ and 1512 cm^−1^ corresponding to PVA’s hydroxyl group, which typically indicates interaction between PVA molecules and Ag nanoparticles [39,41,42,43,44,53]. Additionally, usual peaks representing the C–H group of glycerol (1280 cm^−1^) were found, as well as several peaks from the interval 1170–1134 cm^−1^ corresponding to C–H stretching, C–C stretching mode of PVA, C–O stretching, O–H bending and mode of PVA and glycerol, and C–C stretching mode of PVA and glycerol [39,41,42,43,44,53,54,55]. It should be noted that not only polymer network formation but also C–C bonds scission [55] was present due to the low-dose irradiation of glycerol containing samples, since it is known [39] that radiolysis of PVA with glycerol additives induces formation of silver nanoparticles by PVA and glycerol copolymer degradation and defragmentation. This might be explained by defining the interactions between PVA molecules and Ag nanocrystals as interactions of the OH group and Ag cluster [55]. In such a case, active radicals contributing as silver radiolysis agents are indicated as the PVA and glycerol fragments produced by X-ray exposure of AgGlyPVA composite films. In this case, glycerol in the PVA solution can be considered to play a role not as stabilizer or scavenger, but as the initiator for the formation of Ag nanoparticles in PVA-based films. 

## 4. Conclusions

This study investigated X-ray radiation-induced formation of Ag particles and changes in related optical properties of AgNO_3_—PVA gels and AgNO_3_—PVA gels with glycerol, ethanol, and isopropanol additives. It was found that the amount of the synthesized silver nanoparticles was small but detectable and was dependent on the concentration of the Ag precursor and on the irradiation dose applied. The damping and stabilizing role of glycerol as a scavenger of ${\mathrm{OH}}^{\cdot }$ radicals in AgNO_3_-PVA gels in particle formation process was demonstrated. 

A simple and environmentally friendly method for the formation AgPVA free standing films from AgNO_3_-PVA solutions at room temperature was proposed and realized, and Ag nanoparticles were firstly synthesized in X-ray-irradiated free-standing AgPVA films. The presence of nanoparticles in irradiated films was demonstrated by strong LSPR peaks observed in absorption spectra of films at a wavelength of ~430 nm. 

The intensity of observed LSPR peaks in AgPVA films was slightly dependent on X-ray irradiation dosage up to 4.0 Gy; however, reasonable radiation sensitivity of films was indicated for lower doses (up to 1.0 Gy). The indicated difference between LSPR peak intensity of not-irradiated samples and samples irradiated with the doses from the interval between 0 and 1.0 Gy seems to be a promising result for further investigations related to AgPVA film applications in the medical field of dosimetry.

## Figures and Tables

**Table 1:** Chemical composition of experimental PVA gels.

Materials	Chemical Composition of Samples, wt.%					
	PVA	PVA + Gly	AgPVA1	AgPVA2	AgGlyPVA1	AgGlyPVA2
AgNO_3_	0	0	0.09	0.17	0.09	0.17
(C_2_H_4_O)_n_	20.00	19.51	19.90	19.80	19.51	19.41
C_3_H_8_O_3_	0	2.46	0	0	1.95	1.94
H_2_O	80.00	78.03	80.01	80.03	78.45	78.48

**Table 2:** Chemical composition of as prepared Ag enriched free-standing PVA films.

Material	Chemical Composition of as Prepared Gels Used for Formation of Gel Films, wt.%					
	PVA	AgPVAF1	AgPVAF2	AgGlyPVAF1	AgIsoPVAF1	AgEtaPVAF1
AgNO_3_		0.21	1.01	0.21	0.21	0.21
(C_2_H_4_O)_n_	20.0	19.76	18.92	19.33	19.41	19.35
C_3_H_8_O_3_				2.92		
CH_3_CH_2_OH						2.10
(CH_3_)_2_CHOH					2.53	
H_2_O	80.0	80.03	80.07	77.54	77.85	78.34

## Data Availability

The data in this work are available upon request from the corresponding author.
